# Complete Genome Sequence of *Thermus thermophilus* TMY, Isolated from a Geothermal Power Plant

**DOI:** 10.1128/genomeA.01596-16

**Published:** 2017-02-02

**Authors:** Yasuhiro Fujino, Yuko Nagayoshi, Toshihisa Ohshima, Seiya Ogata, Katsumi Doi

**Affiliations:** a Faculty of Arts and Science, Kyushu University, Motooka, Nishi-ku, Fukuoka, Japan; b Microbial Genetic Division, Institute of Genetic Resources, Faculty of Agriculture, Kyushu University, Hakozaki, Higashi-ku, Fukuoka, Japan; c Department of Biomedical Engineering, Faculty of Engineering, Osaka Institute of Technology, Asahi-ku, Osaka, Japan

## Abstract

*Thermus thermophilus* TMY (JCM 10668) was isolated from silica scale formed at a geothermal power plant in Japan. Here, we report the complete genome sequence for this strain, which contains a chromosomal DNA of 2,121,526 bp with 2,500 predicted genes and a pTMY plasmid of 19,139 bp, with 28 predicted genes.

## GENOME ANNOUNCEMENT

*Thermus* spp. are defined as aerobic, heterotrophic, nonmotile, pigmented, non-spore-forming, Gram-negative rods that can grow in environments over 70°C (1). *Thermus thermophilus* is halotolerant compared with other *Thermus* spp. and has been isolated from hot springs close to the sea and described as saline (2). Strains belonging to* T. thermophilus* are said to be easily distinguished from other species because they can grow on media containing 3% NaCl. *T. thermophilus* strain TMY was isolated from a geothermal power plant located inland in Japan; therefore, it is noteworthy that TMY could not grow in the presence of 3% NaCl, unlike other *T. thermophilus* strains (3). Moreover, strains belonging to *T. thermophilus*, including strain TMY, which was isolated from siliceous deposits, are thought to be involved in silica deposit formation (4–7). Thus, genomic information of strain TMY will be of great help to elucidate these mechanisms. Additionally, genome-scale analysis will provide useful information for industrial applications because members of the genus *Thermus* are of considerable biotechnological interest as sources of thermostable enzymes.

A sample was prepared for sequencing by growing *T. thermophilus* TMY (JCM 10668) overnight at 70°C under aerobic conditions in TM broth, which consisted of 0.4% polypeptone, 0.2% yeast extract, 0.1% NaCl, and 0.1% Castenholz basal salt solution (8). Genomic DNA was isolated by standard cetyltrimethylammonium extraction. The prepared DNA was sequenced using the PacBio RSII platform; 150,292 raw reads resulted in 84,630 quality-filtered trimmed reads yielding 577 Mb, with a mean genome-wide coverage of 256×. The filtered reads were assembled using FALCON version 0.4.0 and resulted in two contig scaffolds. Annotation was performed using the Microbial Genome Annotation Pipeline (MiGAP; http://www.migap.org).

Annotation using the COG, RefSeq, and TrEMBL databases with tRNAscan-SE version 1.23 and additional manual inspection revealed that *T. thermophilus* TMY has a 2,121,526-bp circular chromosome carrying 2,500 predicted genes with a G+C content of 69.0% and a circular 19,139-bp plasmid, pTMY, carrying 28 predicted genes with a G+C content of 67.4%. In total, 47 tRNA genes and six rRNA genes are located in the chromosomal DNA. Interestingly, strain TMY does not have the megaplasmid commonly found in *T. thermophilus* spp. (9). These megaplasmids contain many genes involved in DNA repair and are thought to play an important role in enabling a thermophilic lifestyle; however, strain TMY possesses these genes in its chromosome.

Strain TMY possesses arsenite oxidases (TTMY0009 and TTMY0010), which oxidize arsenite to less toxic arsenate (10). As geothermal water often contains high levels of arsenic (11), silica scales and wastewater from geothermal power plants produce environmental pollution, preventing the efficient utilization of geothermal power. Genomic information implies that strain TMY may be able to perform bioremediation of toxic arsenites.

### Accession number(s).

The results of this whole-genome project have been deposited in DDBJ/EMBL/GenBank under the accession numbers AP017920 and AP017921. The versions described herein are the first versions.
